# Factitious cheilitis: a case report

**DOI:** 10.1186/1752-1947-2-29

**Published:** 2008-01-29

**Authors:** Erdinc Aydin, Ozgur Gokoglu, Gamze Ozcurumez, Hakan Aydin

**Affiliations:** 1 Baskent University Department of Otolaryngology, Ankara, Turkey; 2 Baskent University Department of Psychiatry, Ankara, Turkey; 3 Baskent University Department of Pathology, Ankara, Turkey

## Abstract

**Introduction:**

Factitious cheilitis is a chronic condition characterized by crusting and ulceration that is probably secondary to chewing and sucking of the lips. Atopy, actinic damage, exfoliative cheilitis, cheilitis granulomatosa or glandularis, contact dermatitis, photosensitivity reactions and neoplasia should be considered in the differential diagnosis of crusted and ulcerated lesions of the lip.

**Case presentation:**

We present a 56 year-old female with an ulcerated and crusted lesion on her lower lip. The biopsy showed granulation tissue and associated inflammation but no malignancy. Based on the tissue examination and through clinical evaluation the diagnosis of factitious cheilitis was rendered.

**Conclusion:**

Thorough clinical history, utilization of basic laboratory tests and histopathologic evaluation are required to exclude other diseases and a thoruough psychiatric evaluation and treatment is vital for successful management of these patients.

## Introduction

Self induced disorders may have variable presentations and they may roughly be classified into two groups. The ones with the characteristics of both impulsivity and compulsivity, patients usually acknowledge the self-inflicted nature of the lesions, typical examples of which are pychogenic excoriation (pathological or compulsive skin picking) and trichotillomania (chronic hair pulling) [1]. The other group constitutes abnormal illness behaviors in which patients have concious symptom formation but with motivations that arise from unconcious conflicts ie. Factitious disorders. An important distinction between these two groups of self-inflicted disorders is in the first group patients report their concern and they very much wish to put an end to their behavior while in the second group patients simulate, induce, or aggravate illness, often inflicting painful, deforming, or even-life-threatening injury on themselves primarily to gain the emotional care and attention that comes with playing the role of the sick. Factitious cheilitis, also known as factitious lip crusting, localized crusting or artifactual and exfoliative cheilitis, is a chronic condition characterized by crusting and ulceration [2-4]. It is attributed to self induced trauma such as repetitive bitting, picking or licking of the lips [5]. Preponderance in young women have been reported but it could be seen in any age group and race [2,3,6]. The lesions may be bizarre and hemorrhagic giving clinically a malignant impression [6].

## Case presentation

**Figure 1:**
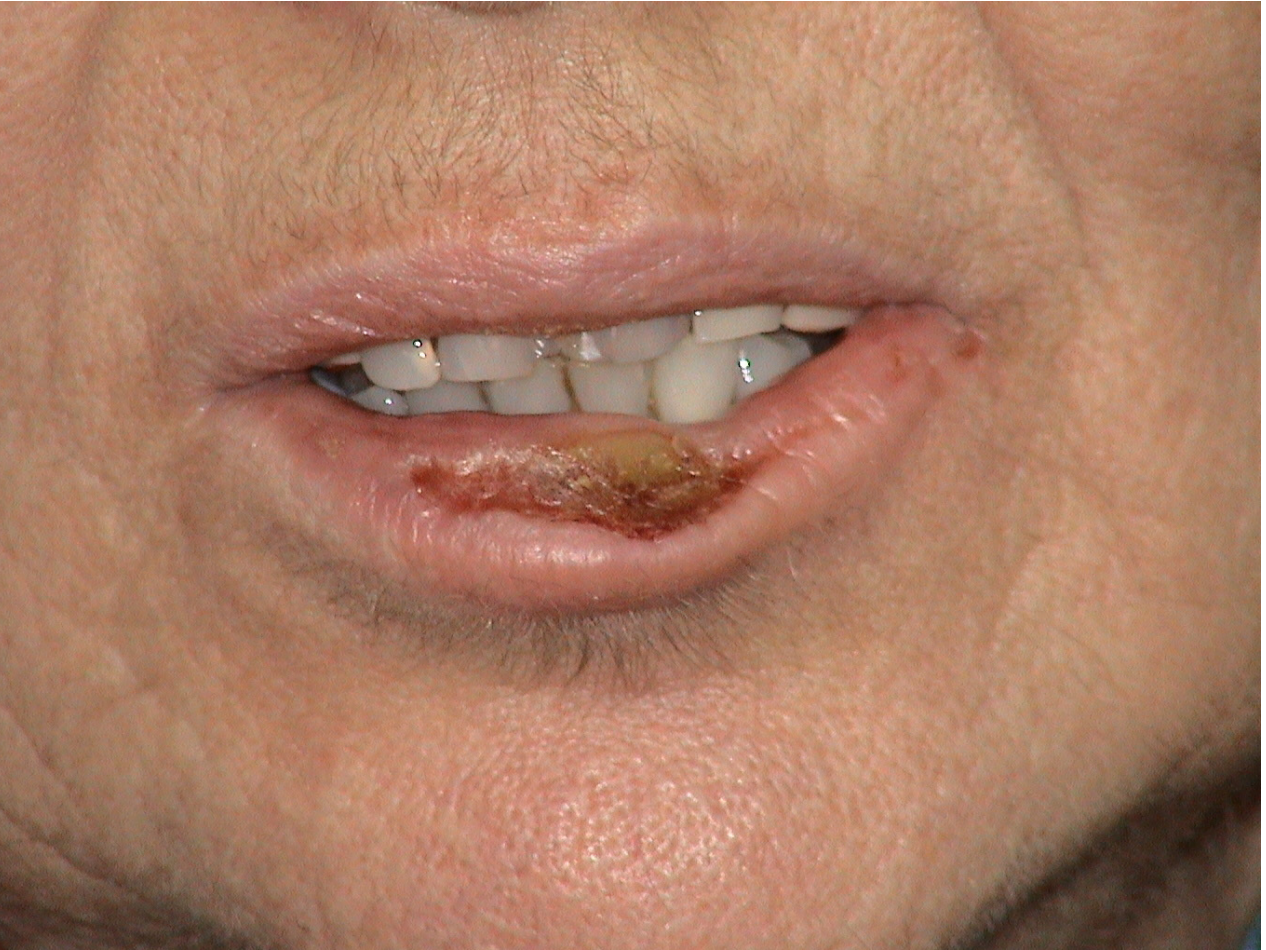
Photograph of an indurated, crusted lesion with central ulceration giving the lip a bitten off appearance.

56 year-old female presented to the otolaryngology department with the chief complaint of a lesion on her lower lip. The lesion was first noticed by the patient 4 years ago and slowly increased in size. Her past medical history was unremarkable. She was not a smoker and there was no weight loss. Physical examination showed a painless, firm, indurated, crusted area of 4 cm with central ulceration giving the lip a bitten off appearance (Figure 1). Otherwise skin and oral mucosa were normal. There were no palpable or ultrasonographically detected lymph nodes. CBC, sedimentation rate, and routine serum chemistries were all within normal limits.

An incisional biopsy that included the normal mucosa and skin was obtained. Histopathological examination showed a non-specific ulcer with inflammation and granulation tissue. There was no cellular atypia in favor of malignancy. Upon detailed history the patient declared her habit of bitting and sucking of the lip that was exacerbated during periods of stress. The patient did not seem gravely concerned about her lesion, nor did she admit to her habit of lip chewing but nevertheless was willing to undergo incisional biopsy. A diagnosis of factitious cheilitis was made and discussed with the patient with a following psychiatry consult. In the meantime, treatment was initiated with petrolatum ointment, chlorhexidine gluconate mouthwash and topical steroids. She refused psychiatric treatment of any kind including the prescribed selective-serotonin reuptake inhibitor (Fluoxetine 20 mg/day). After a year from her first presentation to otolaryngology department her lesion neither healed nor got better.

## Discussion

The exact pathogenesis of factitious chelitis is obscure. Although factitious illness behavior is, by definition, consciously produced, the underlying motivations for the behaviors are largely considered to be unconscious. Despite potentially high stakes, relatively little empirical knowledge is available about the etiology, epidemiology, course and prognosis, and effective treatment of factitious disorders. Methodological problems are inherent in the study of these deceptive patients, as they are difficult to identify, and, when found out, they often flee to avoid charges of fraud [7]. The pyschogenic cause was proposed by Brocq in 1921, who suggested a nervous instability [2]. Schaffer et al. draw attention to borderline personality disorder in factitial dermotosis [3,8]. Coping deficits are widely noted for the etiology of factitious illness behavior. Patients often have immature coping skills, not falling into any current category of personality disorder. This is consistent with observations that many factitious disorder patients come from large families or have been neglected as children, therefore lacking the nurturing conducive to the development of mature coping. On the other hand poor coping may be part of a personality disorder, such as borderline personality or dependent and narcissistic personality traits [5,9].

Our patient strongly opposed psychiatric intervention of any kind which is usually a typical feature of factitious disorder patients. Since she refused further psychiatric evaluation we do not have enough data to have an insight about her past, her personality, her relationship patterns and her coping skills. It is important to note that the psychiatric consultant diagnosed her depression and it is a known fact that factitious illness behavior often occurs in the setting of a loss and emotional turmoil [10]. So the inciting events for factitious illness behavior in our case might be her husband's death which is followed by the departure of her daughters from home. The last blow probably came with the stress of being the sole caregiver for her demented father with whom she lives with for the last five years. Factitious illness behavior can be a maladaptive way of coping with stress and does not necessarily imply an ongoing factitious disorder. This might just be the case for our patient when we consider her living conditions for the last five years together with her untreated depression. Securing the attention of clinicians, family, and friends may be a way of obtaining emotional solace without directly confronting her losses. She might have anger rooted from the obligation of taking care of her demented father on her own and such feelings induce guilt that is extremely hard to cope with most of the time. Especially knowing that one of her daughters is a physician makes these speculations more salient. Her emotional needs and need for recognition and support might only be fullfilled by her factitious cheilitis. Laboratory studies did not support an organic etiology and biopsy findings were not contributory beyond that confirmation of physical findings. Although no laboratory or pathology tests are diagnostic of factitious disorders, they may be useful in demonstrating deception and helping to confirm diagnosis which is usually the case [7]. Some data suggests an associated thyroid abnormality [11,12]. In our patient thyroid function tests were normal.

Atopy, actinic damage, cheilitis, contact dermatititis, photosensitivity reactions and neoplasia should be considered in the differential diagnosis of crusted and ulcerated lesions of the lip [11,13]. Also hypervitaminosis A, lupus erythematosus and licenoid dermatosis have to be ruled out [2,11,13]. Cultures and histopathologic examination are prudent to rule out malignancy and specific infectious etiology. In this case, the patient's history, the negative biopsy findings and a thorough clinical evaluation-including the psychiatry consultation – excluded organic causes.

Psychopharmacological and psychotherapeutic treatments should be used first line according to the diagnosis, depending on the presence of a comorbid DSM-IV (Diagnostic and Statistical Manual of Mental Disorders) Axis I disorder (eg. depression) or a comorbid Axis II disorder (eg. borderline personality). Other than targeting comorbid psychiatric disorders, there is no standard pharmacological treatment for factitious disorder. And one must have to keep in mind that an underlying mood or anxiety disorder that is treatable bodes for a better prognosis, whereas an underlying personality disorder bodes for a poorer prognosis [7]. Topical application of %20 urea, corticosteroids, antibiotics, antifungal agents, petrolatum gels and sunscreens are dermatological treatment agents [2]. However, the response rate is not promising as in our case. Exacerbations are associated with stress and have been shown to regress with psychotherapy and antianxiolytic-antidepressant treatment [2,5,14].

## Conclusion

Factitious cheilitis should be distinguished from infectious cheilitis, contact dermatitis, actinic cheilitis, photosensitivity dermatoses, exfoliative cheilitis, cheilitis glandularis and neoplasia that may look similar on physical exam. Bizarre hemorragic or keratotic crusts should alert the clinician to a possible factitious origin. We noticed that there is an interchangeable usage of exfoliative cheilitis and factitious cheilitis in the literature [5,6,11], however the differentiation of these two has clinical significance. We suggest exfoliative cheilitis is a disorder where patients impulsively/compulsively induce the lesions without any primary goal of attaining the emotional care and attention that comes with the sick role, whereas in factitious disorder the lesions are intentionally produced with the primary goal of the sick role and these patients deny the self inflicted nature of their lesions at all cost. We think that making this differentiation is important in terms of further psychiatric intervention and treatment modalities. As self-inflicted disorders of compulsivity-impulsivity spectrum mostly benefit from selective-serotonin reuptake inhibitors with or without combined low doses of atypical antipsychotics, factitious disorders have to be handled with much more versatile modalities depending on the underlying pathologies. Thorough clinical history, utilization of basic laboratory tests and histopathologic evaluation are required to exclude other diseases and a thoruough psychiatric evaluation and treatment is vital for successful management of these patients.

## Competing interests

The author(s) declare that they have no competing interests.

## Authors' contributions

OG, GO, HA have been involved in drafting the manuscript. HA involved in pathological, GO in the psyhiatric interpretation of the manuscript. EA have involved in revising the manuscript and given the final approval of the version to be published. All authors have read and approved the final manuscript.

## Consent

Written informed consent was obtained from the patient for publication of this Case report and any accompanying images. A copy of the written consent is available for review by the Editor-in-chief of this journal.
